# Actigraphy-based sleep disruption and diurnal biomarkers of autonomic function in paroxysmal atrial fibrillation

**DOI:** 10.1007/s11325-025-03293-4

**Published:** 2025-04-22

**Authors:** Sepideh Khazaie, Lu Wang, Farhad Kaffashi, Mina K. Chung, Catherine M. Heinzinger, David R. Van Wagoner, Kenneth A. Loparo, Harneet K. Walia, Reena Mehra

**Affiliations:** 1 Sleep Disorders Center, Neurological Institute, Cleveland Clinic, Cleveland, OH USA; 2 Department of Quantitative Health Sciences, Cleveland Clinic, Cleveland, OH USA; 3 Institute for Smart, Secure and Connected Systems: ISSACS, Case Western Reserve University, Cleveland, OH USA; 4 The Department of Cardiovascular Medicine, Heart, Vascular and Thoracic Institute, Cleveland Clinic, Cleveland, OH USA; 5 Miami Cardiac and Vascular Institute, Baptist Health South Florida, Miami, FL USA; 6 Pulmonary, Critical Care and Sleep Medicine, Pulmonary and Critical Care Medicine, University of Washington, Seattle, WA USA; 7 American Lung Association Endowed Chair in Pulmonary and Critical Care Medicine Division Head, Pulmonary, Critical Care and Sleep Medicine, University of Washington, 1959 NE Pacific St, Seattle, WA 98195 USA

**Keywords:** Cardiac arrhythmias, Sleep apnea, Continuous positive airway pressure, Heart rate variability

## Abstract

**Introduction:**

Sleep architectural disruption is associated with atrial fibrillation (AF); however, associated autonomic influences remain unclear and it is unknown if this detriment persists during wakefulness. We hypothesize sleep disruption and autonomic dysfunction have diurnal patterning in patients with paroxysmal AF.

**Methods:**

We analyzed data from the Sleep Apnea and Atrial Fibrillation Biomarkers and Electrophysiologic Atrial Triggers (SAFEBEAT) study designed to examine paroxysmal AF and sleep apnea, including simultaneous collection of continuous electrocardiogram monitoring (Heartrak Telemetry^®^) and actigraphy (Actiwatch GTX) for 7–21 days. Heart rate variability (HRV) measures in time-domain (standard deviation of normal-to-normal (NN) intervals (SDNN), coefficient of variation (CV)) and frequency-domain (low frequency power (LFP), high frequency power (HFP)) were used as surrogates of autonomic function and averaged per sleep/wake per day. A linear mixed-effects model assuming compound symmetry correlation structure was used to assess the relationship of HRV with actigraphy-derived sleep data.

**Results:**

The analytic sample (age 60.1 ± 12.0 years, body mass index 32.6 ± 6.7 kg/m2, 36% female, 75% White) included 100 participants with paroxysmal AF. Longer sleep latency was associated with lower HFP during wakefulness (coefficient − 0.0501, *p* = 0.031). Higher sleep efficiency was associated with increased SDNN (coefficient 0.0007, *p* = 0.014) and CV (coefficient 0.0167, *p* = 0.047). Higher arousal index was associated with increased CV (coefficient 0.0166, *p* = 0.007) and LFP (coefficient 0.0232, *p* = 0.003). During sleep, longer average awakenings duration was associated with increased LFP/HFP ratio (coefficient 0.1977, *p* < 0.001) and reduced HFP (coefficient − 0.1338, *p* < 0.001). Significant sleep-wake interactions were observed for sleep latency with HFP (*p* = 0.024), sleep efficiency with SDNN and CV (both *p* < 0.01), WASO with SDNN, CV, and LFP (all *p* < 0.05), and frequency of awakenings with CV and LFP (both *p* < 0.05).

**Conclusions:**

Actigraphy-based measures of sleep disruption were associated with autonomic function alterations exhibiting diurnal variability in paroxysmal AF. Greater overall HRV and parasympathetic modulation were related to better sleep quality. Increased sympathetic activation was associated with sleep fragmentation. Results provide insights into differential autonomic dysfunction related to sleep disruption that may contribute to atrial arrhythmogenesis.

## Introduction

Atrial fibrillation (AF) is the most common sustained cardiac arrhythmia, affecting over 37 million individuals globally [1]. AF is associated with a 5-fold increased risk of stroke and 2-fold increased risk of mortality [2]. Sleep-disordered breathing (SDB), including sleep apnea, is characterized by repetitive apnea and hypopnea events and is highly prevalent in AF, with studies reporting prevalence rates ranging from 21 to 80% [3]. Both SDB and AF are linked to alterations in cardiac autonomic tone, which may contribute to arrhythmogenesis [4].

Heart rate variability (HRV), which reflects oscillations in autonomic inputs to the sinoatrial node, provides a non-invasive assessment of cardiac autonomic function [5]. Reduced HRV signifies increased sympathetic and/or decreased parasympathetic modulation and has been associated with negative cardiovascular outcomes [6]. In AF patients, decreased HRV predicts AF progression, while restoration of sinus rhythm has been associated with improved HRV [7, 8].

While AF and SDB are independently associated with autonomic dysfunction based on HRV analyses [7, 9], the interplay between SDB, HRV, and AF remains incompletely understood. Prior studies have been limited by heterogeneous AF populations, the lack of concurrent HRV and SDB assessments, and inconsistencies in HRV measurement methodologies [10].

Actigraphy using accelerometer data provides an objective evaluation of sleep-wake patterns through continuous activity monitoring [11]. Simultaneous actigraphy and electrocardiogram (ECG) data collection allow for the exploration of dynamic autonomic alterations associated with sleep disruption, with HRV measures derived from the collected ECG data. While subjective reports have linked poor sleep to altered cardiac autonomic control in conditions such as chronic fatigue [12], objective actigraphy-based assessments more accurately quantify sleep disruption. Monitoring for 24 h or longer captures real-world sleep habits, avoiding the limitations of patient-reported data [13]. Actigraphy has been validated against polysomnography for estimating sleep efficiency, wake after sleep onset, and related metrics [14, 15]. This capability enables detailed characterization of sleep-wake patterns and their associations with autonomic function.

Exploring diurnal variations in autonomic function related to SDB may illuminate circadian patterns of arrhythmogenesis in AF. This study aims to characterize the relationships between actigraphy-derived sleep disruption and HRV during sleep versus wakefulness in patients with paroxysmal AF and SDB. We hypothesize that greater sleep disruption correlates with decreased HRV, indicating autonomic dysfunction. Furthermore, we propose that these associations extend into wakefulness, highlighting their potential impact beyond the sleep period.

## Methods

### Study population

This analysis included participants enrolled in the Sleep Apnea and Atrial Fibrillation Biomarkers and Electrophysiologic Atrial Triggers (SAFEBEAT) study (NCT02576587). SAFEBEAT was a prospective cohort study conducted at two academic medical centers from 2012 to 2017. Participants were recruited from cardiology and electrophysiology clinics. Inclusion criteria were age ≥ 55 years, paroxysmal AF defined as self-terminating episodes lasting < 7 days, and an Apnea-Hypopnea Index (AHI) ≥ 15. Exclusion criteria included permanent AF, valvular disease, coronary artery disease, heart failure, hyperthyroidism, prior cardiac surgery or ablation, and other medical comorbidities [16]. The Cleveland Clinic IRB and University Hospitals Case Medical Center IRB each approved this study, and informed consent was obtained from all participants. All methods were performed in accordance with the relevant guidelines and regulations.

### Actigraphy monitoring

Participants underwent continuous wrist actigraphy monitoring (Actiwatch Spectrum, Philips Respironics) for 7–21 days. The actigraph device contains a piezoelectric accelerometer that records limb movements in 3-axes. Participants were instructed to wear the actigraph at all times except when showering or submerged in water. Actigraphy data were analyzed using the Actiware software v6.0.2 in 60-second epochs. Sleep intervals were marked using event markers, sleep diaries, and rest intervals. The following sleep parameters were derived and averaged across all sleep periods [17]: sleep latency, total sleep time, sleep efficiency, wakefulness after sleep onset (WASO), number of awakenings, arousal index, and average awakenings duration. Sleep latency is defined as the time from going to bed to sleep onset; total sleep time is the total time asleep after sleep onset; sleep efficiency is calculated as the total sleep time divided by time in bed multiplied by 100%; WASO represents the total time awake after sleep onset until final awakening; the number of awakenings is the count of episodes where the subject transitions from sleep to wakefulness, each lasting a minimum of 60 s; arousal index is the number of arousals per hour of sleep, indicating sleep fragmentation; and average awakenings duration refers to the average length of these awakenings.

### Electrocardiogram monitoring

Continuous electrocardiogram (ECG) recordings were obtained over the same period as actigraphy using a single-channel telemetry system (Heartrak ECAT, Philips Respironics) with a sampling rate of 250 Hz. Participants were instructed to wear the ECG sensors at all times except during bathing, when actigraphy and ECG monitoring were paused concurrently.

### HRV analysis

HRV measures were derived from normal-to-normal (NN) beat intervals during sinus rhythm on the ECG recordings after excluding segments with noise, ectopy or arrhythmias. Only 5-minute ECG segments meeting stability criteria were analyzed to ensure stationarity [18]. The following HRV measures were examined [19]: In the time-domain, the measures examined included Mean NN (the average of all normal-to-normal intervals), SDNN (standard deviation of normal-to-normal intervals), RMSSD (root mean square of successive differences between normal heartbeats), CV (coefficient of variation of normal-to-normal intervals), and short-term and long-term variability from the Poincaré plot (SD1 and SD2, respectively). In the frequency-domain, we examined low frequency power (LFP), high frequency power (HFP), and the low-to-high frequency power ratio (LHR). Additionally, non-linear measures of variability and complexity, such as the detrended fluctuation analysis (DFA) parameters α1 and α2, were also explored.

HRV indices were computed using custom software developed and implemented in Matlab, and these values were averaged over the total monitoring duration. This analysis was separately conducted for sleep and wake periods as determined by actigraphy.

### Statistical analysis

Participant characteristics were summarized using descriptive statistics. A linear mixed effects model with a compound symmetry covariance structure was used to assess the associations of HRV measures (dependent variables) with actigraphy sleep parameters (independent variables) during sleep and wake periods. Age, sex, race, body mass index (BMI), and relevant medications including anti-hypertensives (ACE inhibitors, ARBs, beta-blockers, calcium channel blockers, diuretics), anti-arrhythmics, anti-depressants, cholesterol-lowering drugs, hypoglycemics, sedatives/sleeping aids were included as covariates. Sleep-wake interactions were examined for each actigraphy index. A p-value < 0.05 was considered statistically significant. All statistical analyses were performed in SAS v9.4 (SAS Institute, Cary NC).

## Results

### Study population

The analytic sample was comprised of100 participants with paroxysmal AF and moderate-severe SDB. Participants underwent actigraphy monitoring for an average of 7.31 ± 2.46 days with an average monitoring duration of 7.16 ± 1.11 h per day. Table 1 summarizes the baseline demographic and clinical characteristics. The mean age was 60.1 ± 12.0 years, 64% were male, 85% were Caucasian, and the mean BMI was 32.6 ± 6.7 kg/m^2^. Hypertension was present in 57% and diabetes in 13%. The majority (83%) were taking antihypertensive medications and 59% were on antithrombotic therapy.

**Table 1:** Characteristics of participants with moderate to severe sleep disordered breathing

	Total (*N* = 100)
Factor	Statistics
Age (years), mean (SD)	60.1 ± 12.0
Body Mass Index (kg/m^2^), mean (SD)	32.6 ± 6.7
Gender N (%)	
Female	36 (36.0)
Male	64 (64.0)
Ethnicity	
Hispanic or Latino/a	1 (1.00)
NOT Hispanic or Latino/a	99 (99.0)
Race	
Caucasian	85 (85.0)
African American	15 (15.0)
High Blood Pressure or Hypertension	57 (57.0)
Diabetes	13 (13.0)
High Blood Cholesterol	62 (62.0)
Heart Attack	3 (3.0)
Stroke	4 (4.0)
Depression	15 (15.0)
Combination of medications: Ace Inhibitors, Antihypertensive, Alpha-2 Blocker, Beta-Blocker, Calcium blocker, Diuretic, Nitrates	83 (83.0)
Ace Inhibitors (Capoten, Vasotec, Zestril, Captopril, Altace, Lisinopril)	27 (27.0)
Angiotensin receptor blocker (Hyzaar, Cozaar, Valsartan)	6 (6.0)
Antiarrhythmic	19 (19.0)
Antiarrhythmic: Class 1a (Na Channel Block, Intermediate) Quinidine, Procainamide, Disoprymide	0 (0.00)
Antiarrhythmic: Class 1b (Na Channel Block, Fast) Lidocaine, Phenytoin, Mexiletine, Tocainide	1 (1.00)
Antiarrhythmic: Class 1c (Na Channel Block, Slow) Flecainide, Propafenone, Moricizine	20 (20.0)
Antiarrhythmic: Class II Beta Blocker	1 (1.00)
Antiarrhythmic: Class III (K + Channel Blocker) Amiodarone, Sotalol, Ibutilide, Dofetilide, Dronedarone	6 (6.0)
Antiarrhythmic: Class IV Slow Channel Blockers (Ca Channel Block) Verapamil, Diltiazem	11 (11.0)
Antiarrhythmic: Class V (Unknown Mechanism) Adenosine, Digoxin, Magnesuim Sulfate	1 (1.00)
Antiarrhythmic: Other	2 (2.0)
Antidepressants (SSRI)	3 (3.0)
Antidepressants, Tricyclic (Elavil, Tofranil, Pamelor)	0 (0.00)
Antidepressants (SNRI) Effexor, Cymbalta, Pristiq	1 (1.00)
Antidepressants, Other (Wellbutrin)	1 (1.00)
Antihypertensive (Hydralazine, Clonidine)	2 (2.0)
Beta-Blocker (Inderal, Lopressor, Tenormin, Corgard, Atenolol, Propranolol)	57 (57.0)
Calcium-channel blocker (Calan, Procardia, Cardizem)	16 (16.0)
Cholesterol-lowering drugs (Mevacor, Pravachol, Zocor, Lipitor)	43 (43.0)
Cholesterol-lowering drugs, Other (Gemfibrozil, Zetia)	9 (9.0)
Diuretic, Loop (Lasix, Furosemide)	3 (3.0)
Diuretic, Thiazide (Hydrochlorothiazide)	17 (17.0)
Diuretic, Other (Aldosterone Antagonist, Spironolactone)	3 (3.0)
Hypoglycemic, Oral (Glyburide, Glucophage)	10 (10.0)
Insulin (Diabetes)	2 (2.0)
Sedative hypnotics (Valium, Xanax, Ativan, Librium)	8 (8.0)
Sleeping Medicine (Ambien, Trazodone)	4 (4.0)

Statistics presented as Mean ± SD, N (column %)

**Table 2:** Pearson correlations of total sleep time and sleep efficiency

Factor	*n*	Pearson Correlation (95% CI)	*p*-value
Same Night Actigraphy vs. PSG			
Total Sleep Time (minutes)	89	0.58 (0.43, 0.71)	**< 0.001**

### Associations between sleep indices and HRV

To assess the validity of actigraphy-derived sleep indices, we examined the correlation between actigraphy and polysomnography (PSG) data in a subset of participants (Table 2). Moderate correlations were observed between same-night actigraphy and PSG measures for total sleep time (TST: *r* = 0.58, 95% CI: 0.43–0.71, *p* < 0.001). Table 3. displays the associations between actigraphy-derived sleep measures and HRV parameters during sleep versus wake periods.

**Table 3:** Associations of heart rate variability with actigraphy sleep measures by sleep and wakefulness

	Sleep		Wakefulness		
Variable	Estimate (95%CI)	*p*-value	Estimate (95%CI)	*p*-value	*P* of interaction
**Association with Actigraphy-Based Sleep Latency**					
*Time Domain Indices*					
MNN	0.0032 (-0.0071,0.0134)	0.55	-0.0061 (-0.0143,0.0021)	0.15	0.15
SDNN	0.0004 (-0.0007,0.0014)	0.49	0.0001 (-0.0007,0.0009)	0.84	0.66
RMSSD	0.0006 (-0.0005,0.0017)	0.26	0.0001 (-0.0008,0.0009)	0.88	0.41
DFA_Alpha1	-0.0044 (-0.0193,0.0105)	0.56	0.0092 (-0.0027,0.0211)	0.13	0.14
DFA_Alpha2	-0.0047 (-0.0244,0.0149)	0.64	0.0025 (-0.0132,0.0181)	0.76	0.56
CV*	0.0073 (-0.0230,0.0387)	0.64	0.0044 (-0.0199,0.0293)	0.72	0.88
SD1*	0.0221 (-0.0217,0.0679)	0.33	-0.0180 (-0.0517,0.0170)	0.31	0.14
SD2*	0.0130 (-0.0193,0.0464)	0.43	-0.0011 (-0.0266,0.0251)	0.94	0.49
SDRatio*	0.0075 (-0.0304,0.0469)	0.70	-0.0169 (-0.0466,0.0136)	0.27	0.30
*Frequency Domain Indices*					
LFP*	0.0135 (-0.0248,0.0534)	0.49	-0.0069 (-0.0370,0.0242)	0.66	0.40
HFP*	0.0314 (-0.0272,0.0934)	0.30	-0.0501 (-0.0934,-0.0048)	***0.031***	***0.024***
LHR*	-0.0030 (-0.0761,0.0758)	0.94	0.0295 (-0.0312,0.0940)	0.35	0.50
**Association with Actigraphy-Based Sleep Efficiency**					
*Time Domain Indices*					
MNN	0.0022 (-0.0031,0.0075)	0.42	0.0062 (0.0006,0.0117)	***0.029***	0.24
SDNN	-0.0003 (-0.0008,0.0003)	0.31	0.0007 (0.0001,0.0013)	***0.014***	***0.004***
RMSSD	-0.0001 (-0.0007,0.0005)	0.74	0.0003 (-0.0003,0.0009)	0.37	0.31
DFA_Alpha1	-0.0013 (-0.0090,0.0064)	0.73	0.0030 (-0.0050,0.0110)	0.46	0.38
DFA_Alpha2	0.0022 (-0.0077,0.0121)	0.67	0.0055 (-0.0047,0.0158)	0.29	0.61
CV*	-0.0151 (-0.0306,0.0005)	0.058	0.0167 (0.0002,0.0335)	***0.047***	***0.002***
SD1*	-0.0108 (-0.0330,0.0119)	0.35	0.0132 (-0.0104,0.0374)	0.27	0.097
SD2*	-0.0121 (-0.0285,0.0045)	0.15	0.0268 (0.0091,0.0447)	***0.003***	***< 0.001***
SDRatio*	-0.0005 (-0.0201,0.0195)	0.96	-0.0142 (-0.0343,0.0063)	0.17	0.28
*Frequency Domain Indices*					
LFP*	-0.0025 (-0.0222,0.0175)	0.80	-0.0168 (-0.0370,0.0037)	0.11	0.26
HFP*	0.0068 (-0.0232,0.0377)	0.66	-0.0163 (-0.0467,0.0151)	0.30	0.23
LHR*	-0.0171 (-0.0550,0.0223)	0.39	-0.0007 (-0.0407,0.0410)	0.97	0.51
**Association with Actigraphy-Based Total Minutes in Bed**					
*Time Domain Indices*					
MNN	-0.0001 (-0.0003,0.0001)	0.19	-0.0000 (-0.0002,0.0001)	0.61	0.54
SDNN	0.0000 (-0.0000,0.0000)	0.20	-0.0000 (-0.0000,0.0000)	0.98	0.32
RMSSD	-0.0000 (-0.0000,0.0000)	0.47	0.0000 (-0.0000,0.0000)	0.60	0.35
DFA_Alpha1	0.0002 (-0.0001,0.0005)	0.13	-0.0000 (-0.0003,0.0002)	0.78	0.17
DFA_Alpha2	-0.0001 (-0.0005,0.0002)	0.49	-0.0002 (-0.0006,0.0002)	0.30	0.79
CV*	0.0006 (0.0000,0.0012)	***0.036***	-0.0002 (-0.0007,0.0004)	0.60	***0.047***
SD1*	-0.0002 (-0.0011,0.0006)	0.57	-0.0001 (-0.0010,0.0007)	0.74	0.86
SD2*	0.0006 (-0.0000,0.0012)	0.055	-0.0001 (-0.0007,0.0005)	0.66	0.073
SDRatio*	-0.0006 (-0.0014,0.0001)	0.074	0.0000 (-0.0007,0.0007)	0.95	0.16
*Frequency Domain Indices*					
LFP*	0.0000 (-0.0007,0.0007)	0.99	0.0010 (0.0003,0.0018)	***0.005***	***0.031***
HFP*	-0.0009 (-0.0020,0.0002)	0.11	-0.0000 (-0.0011,0.0011)	0.94	0.25
LHR*	0.0016 (0.0002,0.0031)	***0.022***	0.0008 (-0.0007,0.0022)	0.29	0.36
**Association with Actigraphy-Based Total Sleep Time**					
*Time Domain Indices*					
MNN	-0.0001 (-0.0003,0.0001)	0.23	-0.0000 (-0.0002,0.0002)	0.85	0.45
SDNN	0.0000 (-0.0000,0.0000)	0.30	0.0000 (-0.0000,0.0000)	0.78	0.57
RMSSD	-0.0000 (-0.0000,0.0000)	0.37	0.0000 (-0.0000,0.0000)	0.52	0.24
DFA_Alpha1	0.0002 (-0.0001,0.0006)	0.12	-0.0000 (-0.0003,0.0003)	0.88	0.20
DFA_Alpha2	-0.0001 (-0.0005,0.0003)	0.62	-0.0002 (-0.0006,0.0002)	0.38	0.77
CV*	0.0006 (-0.0001,0.0012)	0.088	-0.0001 (-0.0007,0.0006)	0.81	0.14
SD1*	-0.0004 (-0.0013,0.0005)	0.40	-0.0001 (-0.0010,0.0009)	0.88	0.60
SD2*	0.0006 (-0.0001,0.0012)	0.10	-0.0000 (-0.0007,0.0007)	0.98	0.21
SDRatio*	-0.0008 (-0.0016,0.0000)	0.058	-0.0000 (-0.0009,0.0008)	0.91	0.18
*Frequency Domain Indices*					
LFP*	0.0000 (-0.0008,0.0008)	0.97	0.0011 (0.0002,0.0019)	***0.011***	0.056
HFP*	-0.0010 (-0.0022,0.0002)	0.11	-0.0001 (-0.0014,0.0011)	0.83	0.30
LHR*	0.0018 (0.0002,0.0034)	***0.026***	0.0008 (-0.0008,0.0024)	0.31	0.36
**Association with Actigraphy-Based Wake After Sleep Onset**					
*Time Domain Indices*					
MNN	-0.0007 (-0.0017,0.0003)	0.16	-0.0008 (-0.0018,0.0002)	0.10	0.86
SDNN	0.0001 (-0.0000,0.0002)	0.088	-0.0001 (-0.0002,0.0000)	0.21	***0.019***
RMSSD	0.0000 (-0.0001,0.0001)	0.74	-0.0000 (-0.0001,0.0001)	0.83	0.66
DFA_Alpha1	0.0005 (-0.0009,0.0020)	0.47	-0.0006 (-0.0020,0.0008)	0.39	0.21
DFA_Alpha2	-0.0012 (-0.0031,0.0006)	0.20	-0.0012 (-0.0031,0.0006)	0.19	0.99
CV*	0.0042 (0.0012,0.0071)	***0.005***	-0.0021 (-0.0050,0.0009)	0.17	***< 0.001***
SD1*	0.0017 (-0.0025,0.0059)	0.43	-0.0018 (-0.0059,0.0024)	0.41	0.20
SD2*	0.0034 (0.0003,0.0065)	***0.031***	-0.0031 (-0.0061,0.0000)	0.052	***0.001***
SDRatio*	-0.0009 (-0.0045,0.0028)	0.64	0.0016 (-0.0020,0.0053)	0.39	0.29
*Frequency Domain Indices*					
LFP*	0.0001 (-0.0036,0.0037)	0.97	0.0055 (0.0019,0.0092)	***0.003***	***0.021***
HFP*	-0.0027 (-0.0083,0.0029)	0.34	0.0022 (-0.0034,0.0078)	0.45	0.17
LHR*	0.0059 (-0.0014,0.0133)	0.11	0.0029 (-0.0044,0.0102)	0.44	0.52
**Association with Actigraphy-Based Number of Awakenings**					
*Time Domain Indices*					
MNN	-0.0027 (-0.0068,0.0013)	0.19	-0.0027 (-0.0067,0.0013)	0.19	0.99
SDNN	0.0003 (-0.0001,0.0007)	0.20	0.0001 (-0.0003,0.0005)	0.77	0.42
RMSSD	0.0002 (-0.0002,0.0007)	0.30	0.0001 (-0.0003,0.0005)	0.63	0.65
DFA_Alpha1	0.0010 (-0.0049,0.0068)	0.75	-0.0021 (-0.0079,0.0037)	0.48	0.41
DFA_Alpha2	-0.0001 (-0.0077,0.0074)	0.97	-0.0009 (-0.0085,0.0066)	0.81	0.87
CV*	0.0166 (0.0044,0.0289)	***0.007***	0.0003 (-0.0116,0.0124)	0.96	***0.034***
SD1*	0.0143 (-0.0031,0.0319)	0.11	-0.0001 (-0.0171,0.0173)	0.99	0.19
SD2*	0.0136 (0.0008,0.0266)	***0.037***	-0.0028 (-0.0154,0.0099)	0.66	***0.041***
SDRatio*	0.0015 (-0.0135,0.0167)	0.84	0.0027 (-0.0122,0.0179)	0.72	0.90
*Frequency Domain Indices*					
LFP*	-0.0050 (-0.0199,0.0101)	0.51	0.0232 (0.0080,0.0387)	***0.003***	***0.003***
HFP*	0.0067 (-0.0162,0.0301)	0.57	-0.0012 (-0.0239,0.0219)	0.91	0.59
LHR*	-0.0006 (-0.0300,0.0298)	0.97	0.0196 (-0.0103,0.0505)	0.20	0.29
**Association with Actigraphy-Based Average Awakening Length**					
*Time Domain Indices*					
MNN	0.0002 (-0.0136,0.0141)	0.97	-0.0060 (-0.0196,0.0076)	0.39	0.48
SDNN	0.0008 (-0.0006,0.0021)	0.29	-0.0021 (-0.0034,-0.0007)	***0.003***	***0.002***
RMSSD	-0.0008 (-0.0023,0.0006)	0.27	-0.0008 (-0.0023,0.0006)	0.25	0.99
DFA_Alpha1	0.0064 (-0.0136,0.0263)	0.53	-0.0068 (-0.0265,0.0128)	0.49	0.30
DFA_Alpha2	-0.0257 (-0.0517,0.0003)	0.053	-0.0105 (-0.0360,0.0150)	0.42	0.37
CV*	0.0193 (-0.0216,0.0619)	0.36	-0.0465 (-0.0842,-0.0073)	***0.021***	***0.010***
SD1*	-0.0507 (-0.1048,0.0068)	0.083	-0.0456 (-0.0992,0.0112)	0.11	0.89
SD2*	0.0256 (-0.0179,0.0711)	0.25	-0.0534 (-0.0929,-0.0121)	***0.012***	***0.004***
SDRatio*	-0.0638 (-0.1106,0.0146)	***0.012***	0.0196 (-0.0305,0.0723)	0.45	***0.009***
*Frequency Domain Indices*					
LFP*	0.0418 (-0.0106,0.0970)	0.12	0.0308 (-0.0202,0.0845)	0.24	0.75
HFP*	-0.1338 (-0.1988,0.0636)	***< 0.001***	0.0443 (-0.0328,0.1276)	0.27	***< 0.001***
LHR*	0.1977 (0.0821,0.3257)	***< 0.001***	-0.0157 (-0.1092,0.0877)	0.76	***0.002***

Abbreviations used in the table:

· MNN: Mean NN Interval

· SDNN: Standard Deviation of NN Intervals

· RMSSD: Root Mean Square of Successive Differences

· CV: Coefficient of Variation

· SD1: Standard Deviation of Short-Term Heart Rate Variability

· SD2: Standard Deviation of Long-Term Heart Rate Variability

· SDRatio: Ratio of SD1 to SD2

· LFP: Power in the Low Frequency Range (0.04–0.15 Hz)

· HFP: Power in the High Frequency Range (0.15–0.4 Hz)

· LHR: Ratio of Low Frequency Power to High Frequency Power

### Associations between sleep latency and HRV

Longer sleep latency, reflecting greater difficulty falling asleep, was associated with reduced HFP during wakefulness (coefficient − 0.0501, 95% CI [-0.0934,-0.0048], *p* = 0.031). However, no relationship was observed between sleep latency and HFP during sleep. A significant sleep-wake interaction was present for the association between sleep latency and HFP (*p* = 0.024), indicating this relationship differed between sleep versus wake periods.

### Associations between sleep efficiency and HRV

Higher sleep efficiency was associated with increased SDNN, CV and Poincaré plot SD2 during wakefulness (SDNN coefficient 0.0007, 95% CI [0.0001,0.0013], *p* = 0.014; CV coefficient 0.0167, 95% CI [0.0002,0.0335], *p* = 0.047; SD2 coefficient 0.0268, 95% CI [0.0091,0.0447], *p* = 0.003). No relationships were observed between sleep efficiency and HRV metrics during sleep. Significant sleep-wake interactions were present for SDNN and CV (both *p* < 0.01), denoting that these associations differed between sleep and wake periods.

### Associations between total sleep time and HRV

In contrast to other sleep parameters, total sleep time did not demonstrate significant associations with any HRV measures during either sleep or wakefulness. No sleep-wake interactions were statistically significant for the relationships between total sleep time and all HRV indices.

### Associations between WASO and HRV

Higher WASO, was associated with increased coefficient of variation (CV) and Poincaré plot SD2 during sleep (CV coefficient 0.0042, 95% CI [0.0012,0.0071], *p* = 0.005; SD2 coefficient 0.0034, 95% CI [0.0003,0.0065], *p* = 0.031). WASO was also associated with increased low frequency power (LFP) during wakefulness (coefficient 0.0055, 95% CI [0.0019,0.0092], *p* = 0.003). Significant sleep-wake interactions were present for CV, SD2 and LFP (all *p* < 0.05), signifying that these relationships differed between sleep and wake periods.

### Associations between number of awakenings and HRV

More frequent nocturnal awakenings were associated with increased CV during sleep (coefficient 0.0166, 95% CI [0.0044,0.0289], *p* = 0.007). The number of awakenings was also associated with increased LFP during wakefulness (coefficient 0.0232, 95% CI [0.0080,0.0387], *p* = 0.003). Significant sleep-wake interactions were observed for both CV and LFP (both *p* < 0.05), denoting differential relationships by sleep-wake state.

### Average awakening duration

Longer average awakening duration was associated with increased LHR and decreased HFP during sleep (LHR: coefficient 0.1977, 95% CI [0.0821,0.3257], *p* < 0.001; HFP: coefficient − 0.1338, 95% CI [-0.1988, -0.0636], *p* < 0.001). In contrast, longer awakening length was associated with reduced SDNN, CV and SD2 in wakefulness. Significant sleep-wake interactions were present for each of these HRV measures (all *p* < 0.01).

**Fig. 1:**
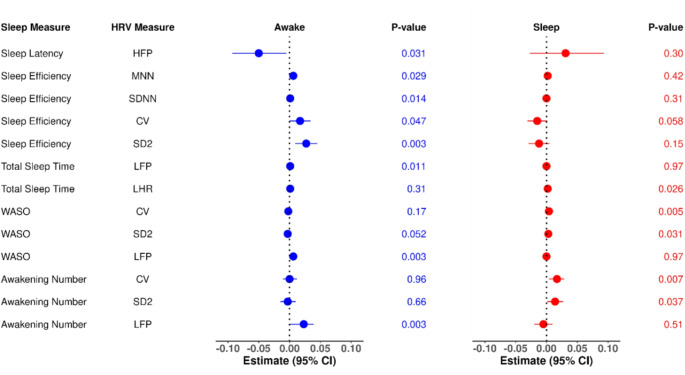
Forest plot illustrating the differential associations of actigraphy-derived sleep measures with heart rate variability indices during sleep (red) versus wakefulness (blue). The regression coefficients and p-values are displayed for each sleep parameter

**Fig. 2:**
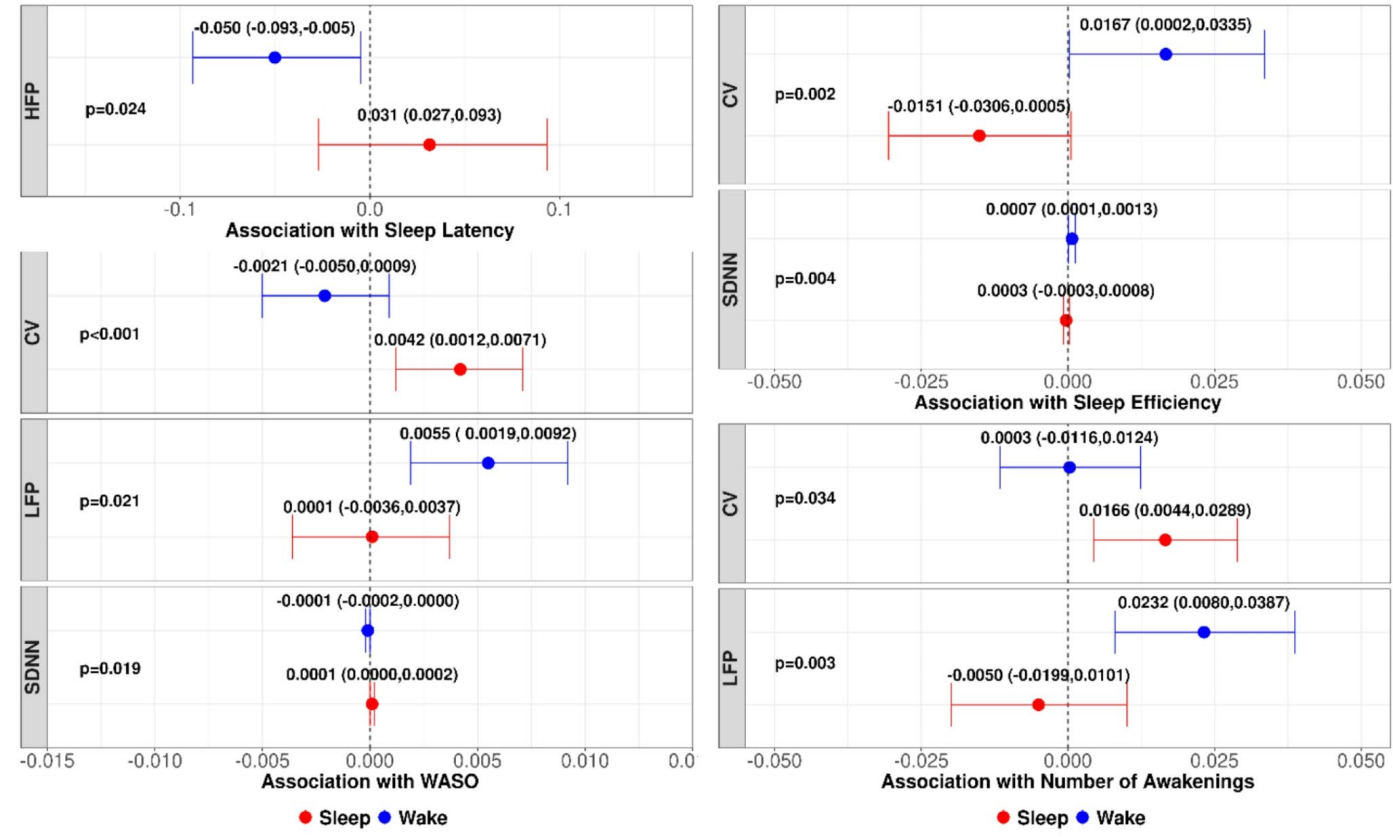
Interaction plots displaying the sleep-wake interactions for the relationships between select actigraphy sleep measures and heart rate variability parameters. The regression lines illustrate the differing slopes between sleep (red) and wakefulness (blue). Significant sleep-wake interactions (*p* < 0.05) were present for sleep latency with high frequency power, sleep efficiency with SDNN and CV, wake after sleep onset with SDNN, CV, and low frequency power, and number of awakenings with CV and low frequency power

Figure 1 highlights the differential associations between specific sleep indices and HRV parameters during sleep versus wakefulness. Figure 2 illustrates the sleep-wake interactions for HRV relationships with sleep latency, sleep efficiency, WASO and number of awakenings. Objective measures of sleep disruption demonstrated significant associations with alterations in HRV indicative of autonomic dysfunction. Notably, these relationships varied between sleep and wake periods, with more consistent HRV changes observed during wakefulness.

## Discussion

In this study of individuals with moderate to severe OSA and paroxysmal AF, we observed significant associations between actigraphy-derived measures of sleep disruption and alterations in HRV indicative of autonomic dysfunction in patients with paroxysmal AF and sleep disordered breathing. Importantly, these relationships exhibited diurnal variation, with greater HRV changes observed during wakefulness compared to sleep. Our findings of greater degree of sleep disruption associated with HRV alterations more pronounced during wakefulness align with known circadian patterns of autonomic activity, which could provide insights into daily cycles of AF onset in the setting of at least a moderate degree of sleep disordered breathing.

The associations of poorer sleep quality with reduced HRV suggest that sleep disturbances may promote autonomic imbalance in AF patients. Possible mechanisms include activation of inflammatory pathways and oxidative stress by sleep deprivation that modulate cardiac autonomic function [20, 21]. Sleep fragmentation with frequent arousals can also directly provoke surges in sympathetic activity and vagal withdrawal [22]. Shortened and fragmented sleep can induce inflammatory cytokines like C-reactive protein (CRP), interleukin-6 (IL-6) and tumor necrosis factor-alpha (TNF-alpha) [23, 24], which have been associated with reduced HRV through effects on the sinoatrial node [25]. Additionally, CRP levels are elevated during daytime compared to nocturnal AF episodes [26, 27], further linking inflammatory pathways to circadian patterns of arrhythmogenesis. Our findings thus align with evidence that inflammatory processes induced by sleep disruption may alter cardiac autonomic function, reflected in the HRV changes observed. This also indicates that the effects of sleep impairment extending beyond the sleep period into wakefulness may be mediated by sustained sympathetic excitation and impaired vagal recovery after sleep disruption [26].

Our observations of sleep disruption and altered HRV during wake periods corroborate established circadian rhythms in autonomic functions. These insights potentially elucidate the daily fluctuations in AF occurrence. Sympathetic nervous system tone typically peaks during daytime waking hours, while parasympathetic activity is more prominent at night during sleep. This variation may offer additional insights into the sympathetic and parasympathetic balance in different sleep-wake schedules [5, 28]. This circadian variation in autonomic balance has been associated with increased propensity for ventricular arrhythmias in the morning period of time [29]. Similarly, in AF patients, the persistent HRV changes we observed during daytime wakefulness following sleep disruption suggest autonomic dysfunction is not limited solely to the sleep period. The observation of more prominent HRV changes during wakefulness has implications for understanding circadian patterns of arrhythmogenesis in AF, though further study relating these HRV metrics to timing of arrhythmia onset is needed. Overall, our findings highlight the interplay between sleep disturbances and autonomic dysregulation showing diurnal variability in this population.

Our results provide novel insights into the diurnal autonomic dysfunction associated with sleep impairment in patients with AF and sleep apnea. However, several limitations should be acknowledged.

First, the microlongitudinal study design, while allowing for repeated measures, cannot establish causality. Confounding factors that were not measured may also influence the observed relationships. Establishing a causal link between HRV alterations and the initiation or progression of AF remains an area for future research. Prospective interventional studies, such as those utilizing continuous positive airway pressure (CPAP) therapy, could provide more direct evidence of these relationships.

Second, while actigraphy offers practical insights for large-scale and longitudinal studies due to its convenience, it lacks the detailed resolution of PSG. PSG provides comprehensive information on sleep architecture, including REM and NREM stages, which are critical for understanding sleep abnormalities. To enhance the reliability of findings, future studies could consider multi-night or repeated longitudinal PSG measures.

Third, while HRV serves as a valuable non-invasive surrogate for autonomic function, it does not fully capture the intricate balance between sympathetic and parasympathetic activity. More direct measures, such as microneurography, provide higher precision in assessing autonomic nervous system alterations. However, the invasive nature of these techniques and their inability to continuously monitor diurnal variations limit their feasibility within our study design. To address these limitations, future research should consider integrating HRV with catecholamine biomarkers (e.g., plasma norepinephrine and epinephrine levels) or employing direct sympathetic nerve activity assessments such as microneurography. This multimodal approach would enhance the understanding of how sleep disruption contributes to autonomic dysfunction and cardiovascular disease progression.

Fourth, the study does not account for critical mediators like inflammation, oxidative stress, or hormonal influences, which play a pivotal role in modulating cardiovascular and autonomic health. These pathways are particularly relevant in the context of AF and sleep disorders. Future studies should aim to include these biomarkers to better elucidate their contributions to the observed associations.

Finally, the analysis primarily focuses on HRV without addressing clinical outcomes such as AF recurrence rates, symptom burden, or responses to treatment. Investigating these outcomes in conjunction with HRV measures would provide important translational insights and reinforce the clinical relevance of the findings. For instance, examining the effects of interventions like CPAP therapy on HRV and AF recurrence could bridge the gap between mechanistic insights and patient outcomes.

Despite these limitations, the study has notable strengths, including the use of continuous objective sleep monitoring and prolonged ECG recording over multiple days. This comprehensive approach enables a detailed and accurate assessment of sleep patterns and their impact on cardiovascular health. Moreover, the robust HRV analytics employed offer sophisticated insights into the complex interplay between sleep disruption and cardiac health, providing a strong foundation for future research in this field.

Our findings are internally consistent across multiple HRV measures and align with experimental evidence indicating causal effects of sleep impairment on autonomic function [30–32] Interventional studies of total and partial sleep deprivation have demonstrated reductions in HRV metrics including a range of spectral and frequency-based measures [33]. This supports sleep disruption as a potential contributor rather than merely a biomarker of autonomic imbalance and underlying cardiovascular dysfunction.

Our results suggest utility for actigraphy-based sleep assessment to identify AF patients who may be at risk for autonomic dysfunction and associated outcomes, e.g. progression of arrhythmia burden. Targeting sleep quality improvement as a modifiable risk factor could potentially optimize autonomic function in this population. This is supported by studies showing alterations in HRV after treatment of sleep apnea with positive airway pressure [34]. Further research is warranted to determine whether optimizing sleep quality can improve cardiac autonomic control and reduce arrhythmia susceptibility in AF patients with sleep disorders.

In conclusion, indices of worsened sleep disruption demonstrated significant associations with HRV alterations indicating autonomic dysfunction in patients with paroxysmal AF and sleep apnea. Importantly, these associations were more consistently observed during wakefulness compared to sleep. Our findings suggest that sleep disruption may contribute to diurnal variability in cardiac autonomic function, which could potentially influence circadian patterns of AF. Additional studies exploring the arrhythmogenic mechanisms associated with sleep-autonomic interactions in AF are necessary.

## Data Availability

The data generated during this study are available from the corresponding author upon reasonable request.
